# Electrical Energy Storage from Low-Grade Heat Using Reduced Graphene Oxide–Carbon Nanotube Composite Materials

**DOI:** 10.3390/ma18204807

**Published:** 2025-10-21

**Authors:** Zhe Yang, Yijia Xu, Shuocheng Sun, Yujia Zhang, Xiaolu Li, Yan Zhao, Xusheng Hao, Caige Xue, Dening Guo, Jia Li, Jiale Wang

**Affiliations:** 1 Henan International Joint Laboratory of New Civil Engineering Structure, School of Intelligent Construction and Civil Engineering, Luoyang Institute of Science and Technology, Luoyang 471023, China; 13468653507@163.com (S.S.); 18695991194@163.com (Y.Z.); lixiaolueven@126.com (X.L.); yan6327zhao@163.com (Y.Z.); 15225004356@163.com (C.X.); 13233969178@163.com (D.G.); 16637936893@163.com (J.L.); 15227717981@163.com (J.W.); 2 Henan Engineering Research Center of Water Quality Safety in the Middle-Lower Yellow River, Henan Green Technology Innovation Demonstration Base, Luoyang Institute of Science and Technology, Luoyang 471023, China; 3 Henan Key Laboratory of Green Building Materials Manufacturing and Intelligent Equipment, Luoyang Institute of Science and Technology, Luoyang 471023, China; 4 Leeds Joint School, Southwest Jiaotong University, Chengdu 611756, China; 13468705627@163.com

**Keywords:** RGO-CNTs composite, electric double layer, low-grade heat, thermoelectric conversion cell, thermoelectric coefficient

## Abstract

The conversion of low-grade heat into storable electrical energy using nanoporous carbon materials represents an efficient energy harvesting strategy. In this study, a reduced graphene oxide (RGO) and carbon nanotube (CNT) composite with a rich microporous structure was synthesized. A symmetrical thermoelectric cell was constructed to harvest thermal energy. The application of a temperature difference (Δ*T*) generated a stable equilibrium voltage (*U*_s_), which scaled linearly with Δ*T*. The resulting thermoelectric coefficient (*U*_s_/Δ*T*) increased markedly with the carbon nanotube (CNT) content, underscoring the effectiveness of CNT incorporation for improving thermoelectric properties. It also shows a non-monotonic dependence on KCl concentration, first increasing and then decreasing, with a maximum value of 4.17 mV/°C achieved in 0.1 M KCl using the RGO-5%CNTs electrode. When connected to an external load, the discharge voltage and current decay rapidly before stabilizing within seconds. Circuit analysis reveals that the incorporation of CNTs reduces internal resistance and increases the equivalent capacitance. Although instantaneous discharge power declines quickly, the addition of CNTs elevates its initial value and slows the decay rate. Both the average output power and thermoelectric conversion efficiency improve with increasing Δ*T* and are further enhanced at higher CNT content. Overall, the RGO-CNT composite demonstrates significantly superior thermoelectric performance compared to pure RGO.

## 1. Introduction

Carbon nanotubes (CNTs) are renowned for their exceptional mechanical, electrical, and thermal properties, stemming from their well-defined atomic structure. For instance, CNTs are among the strongest materials in the world, with a tensile modulus exceeding 1 TPa and a tensile strength over 100 GPa [1]. They also exhibit exceptional thermal and electrical conductivity, reaching up to 3500 W/(m∙K) and 2 × 10^7^ S/m, respectively [2]. These extraordinary properties, which arise from the graphitic arrangement of carbon atoms and their unique structure, make CNTs highly suitable for diverse applications in energy storage, electronics, structural composites, and biomaterials [3].

Graphene has attracted significant attention in recent years due to its unique structure and exceptional properties, leading to promising applications across numerous industries. It is renowned for being the thinnest known material while also exhibiting extreme mechanical strength, with a tensile strength of ~130 GPa—roughly 200 times greater than that of steel [4,5]. Its functional properties are equally impressive, including a high thermal conductivity of 5000 W/(m·K), excellent charge carrier mobility of 15,000 cm^2^/(V·s) at room temperature, and a white-light transmittance of ~95% for a single layer [6,7,8]. Graphene also possesses a large theoretical specific surface area of 2630 m^2^/g [9], coupled with high flexibility and ease of functionalization [10]. This capacity for functionalization enhances its dispersion and tailors its properties, enabling advanced applications in fields such as optoelectronics, catalysis, energy storage, sensing, and actuation [5,10,11].

In the rapidly evolving renewable energy sector, the large-scale deployment of intermittent clean energy sources such as solar and wind power remains constrained by limitations in energy storage technology [12,13,14]. At the same time, a substantial amount of low-grade waste heat generated from industrial processes (generally defined as thermal energy below 230 °C) continues to be underutilized [15,16]. Within the current energy system, approximately 75% of fossil fuel consumption is dedicated to heating and thermal processes, with over 60% of the input energy ultimately released into the environment as waste heat, representing a major efficiency loss [17]. This reality stands in stark contrast to China’s strategic targets of achieving carbon peak and carbon neutrality, underscoring the urgency of improving energy utilization [18,19]. Although several technologies have been developed for low-grade waste heat recovery, multiple challenges persist [20,21]. Conventional heat engines are limited by Carnot efficiency, which restricts their effectiveness in converting low-temperature heat sources [22]. Heat pumps, while technically viable, face barriers to widespread adoption due to their high cost [23]. Moreover, spatiotemporal mismatches between heat supply and demand result in significant volumes of low-grade waste heat remaining untapped [24,25]. Addressing these issues is crucial not only for enhancing energy efficiency in the ‘last mile’ of energy use, but also for fostering interdisciplinary innovation spanning materials science, thermodynamics, and systems engineering. Such advances will provide essential support for building a low-carbon, high-efficiency energy system for the future [26,27].

The thermoelectric conversion cell, which operates on the Electric Double Layer (EDL) principle and utilizes nanoporous carbon materials, represents an emerging technology for recovering low-grade waste heat. Its fundamental mechanism relies on the large specific surface area of nanoporous carbon to induce a potential difference between electrodes. This is achieved through temperature-gradient-driven selective adsorption and desorption of ions in the electrolyte, enabling the direct conversion of thermal energy into electrical energy. At present, research in this field—both domestically and internationally—remains in the foundational laboratory stage, yet it shows a clear trend of rapid development through mutual reference and knowledge sharing across studies. International research began even earlier, with Xi Chen’s team revealing the thermal response mechanisms of electrolyte solution transport in carbon nanotubes as well as the thermal response mechanisms of surface potential in carbon nanotubes [28,29]. For instance, German researchers systematically investigated the thermal response behavior of ionic liquids in carbon materials with tailored pore structures, providing deeper insights into the pore-ion matching mechanism [30]. A team from the Georgia Institute of Technology demonstrated that incorporating multi-walled carbon nanotubes into the electrolyte reduces mass transport resistance, thereby enhancing the power output of thermally chargeable capacitors [31]. Research efforts in China have also kept pace with international advances, with notable progress in material optimization and prototype device performance in recent years. For example, a group from Shenzhen University improved the electrochemical performance of devices by designing hierarchically porous carbon materials (e.g., activated carbon and carbon nanospheres) to synergistically enhance ion transport dynamics [32]. Meanwhile, researchers at Northeast Electric Power University reported carbon materials derived from metal–organic frameworks (MOFs) that enable high output power density even under small temperature differences [33]. Other teams have explored asymmetric electrode configurations, such as pairing carbon materials with distinct pore sizes, leveraging capacitive disparities between hot and cold ends for improved output efficiency [34]. Despite these advances, the technology still faces several common challenges, including low energy conversion efficiency (typically below 1%), insufficient output power for practical applications, limited long-term cycling stability, and scalability issues for large-scale integration.

In parallel to solid-state thermoelectrics, thermoelectrochemical cells (TECs), also known as thermocells, have emerged as a promising alternative for harvesting low-grade thermal energy [35]. Unlike conventional thermoelectrics that rely on the Seebeck effect in semiconductors, TECs utilize the temperature dependence of electrochemical redox potentials in an electrolyte/electrode system [36]. Typically, a TEC consists of two electrodes immersed in an electrolyte containing a redox couple (e.g., Fe(CN)_6_^3−^/^4−^, I^−^/I_3_^−^). When a temperature gradient (Δ*T*) is applied between the electrodes, it induces a difference in the electrochemical potentials, generating a net voltage output due to the entropic change in the redox reactions [37]. The Seebeck coefficient of TECs, which quantifies their performance, can be an order of magnitude larger than that of inorganic semiconductors [38]. However, the widespread application of TECs faces several challenges, including relatively low power density and long-term stability issues. Recent research efforts have been directed towards developing advanced electrode materials, such as porous carbons, to increase the electrochemically active surface area and enhance the rate of redox reactions, thereby boosting the output power [37,39]. In this context, carbon nanomaterials like graphene and carbon nanotubes are particularly attractive due to their high electrical conductivity, large specific surface area, and tunable surface chemistry. Building upon this foundation, this study designed and prepared self-assembled RGO-CNT composite, with a focus on developing high-efficiency thermoelectric conversion cells.

## 2. Method

**Prepare graphene oxide.** Firstly, a graphene oxide (GO) suspension was prepared using an improved Hummers method [40]. Specifically, 3 g of graphite flakes (750 mesh) and 1.5 g of sodium nitrate were gradually added to 75 mL of concentrated sulfuric acid maintained at 0–5 °C in an ice bath under gentle stirring. After one hour, 12 g of potassium permanganate was added in three portions at 10 min intervals, and stirring continued for 2 h. The beaker was then removed from the ice bath and left at room temperature for 1 h, followed by stirring at 35 °C for 1 h, which yielded a brown suspension. This suspension was subsequently cooled in an ice bath and slowly diluted with 135 mL of deionized water. The mixture was stirred at 90 °C for 30 min, after which the reaction was terminated by adding 10 mL of 30% hydrogen peroxide along with 82 mL of deionized water, resulting in a bright yellow graphene oxide dispersion. After cooling, the product was washed repeatedly with 1 M hydrochloric acid and deionized water until no chloride ions were detected, and then centrifuged with deionized water to obtain the final GO suspension.

**Preparation of graphene.** A suspension containing 300 mg of GO was diluted with deionized water and centrifuged at 10,000 rpm for 10 min. The supernatant was discarded to obtain a concentrated GO paste. This paste was then redispersed in 100 mL of N, N-dimethylformamide (DMF) added gradually, followed by thorough stirring for 30 min. Next, 50 mL of ethylenediamine was added at room temperature. The resulting mixture was then transferred to a beaker and heated to 85 °C in a water bath with continuous stirring for 12 h. After the reaction, the product was dialyzed against 200 mL of 20% ethanol solution for 12 h at room temperature. Finally, the sample was washed repeatedly with deionized water until the pH of the wash solution reached approximately 7, yielding an aqueous dispersion of reduced graphene oxide (RGO).

**Figure 1:**
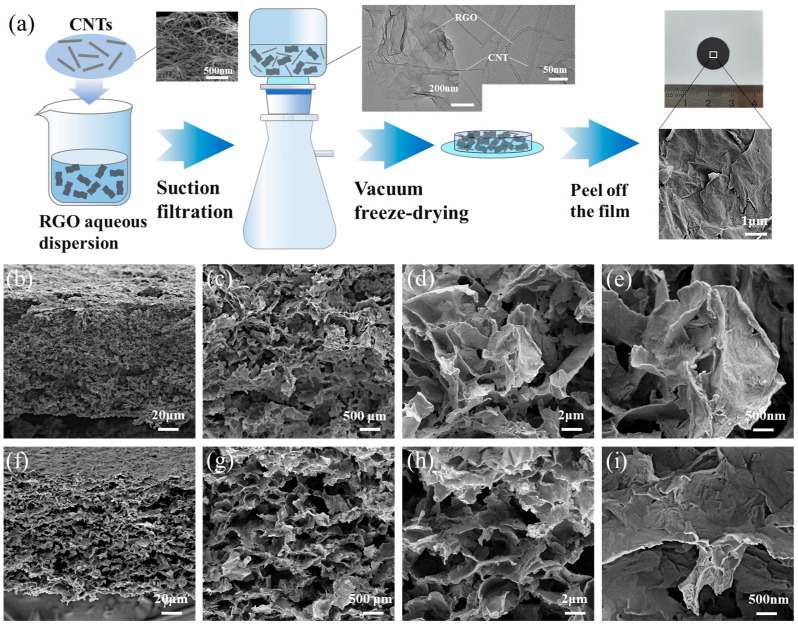
(**a**) Preparation process of the RGO-CNTs composite material. The inset in the middle of (**a**) is a TEM image of the RGO and CNTs mixture. (**b**–**e**) Cross-sectional micrographs of the RGO-5%CNTs composite material. (**f**–**i**) Cross-sectional micrographs of the RGO.

**Preparation of reduced graphene oxide–carbon nanotubes materials.** A method combining vacuum filtration and freeze-drying was employed to prepare reduced graphene oxide–carbon nanotubes (RGO-CNTs) composite materials for thermoelectric conversion cell electrodes. The preparation procedure is illustrated in Figure 1a. First, a 1 mg/mL aqueous dispersion of RGO was prepared, into which single-walled carbon nanotube powder (length 5–20 μm) was added. After 30 min of ultrasonic treatment, the mixture was subjected to vacuum filtration using a cellulose filter membrane assembly, yielding a moist composite film. The TEM image of the RGO and CNTs mixture is shown as an inset in the middle part of Figure 1a. To prevent moisture loss and avoid restacking of RGO sheets, the film was immediately transferred to a vacuum freeze dryer (Gipp LGJ-10A-50, Shanghai, China) pre-cooled to −65 °C. Following 2 h of freezing, the vacuum pump was activated while the refrigeration system remained operational to trap sublimated water vapor. The temperature was then gradually increased until complete dehydration was achieved, resulting in a dry RGO-CNTs composite. It is worth noting that composites with different CNT mass ratios were obtained by adjusting the amount of CNT powder added. The samples are denoted as RGO, RGO-1%CNTs, RGO-3%CNTs, and RGO-5%CNTs, corresponding to CNT contents of 0%, 1%, 3%, and 5%, respectively. Compositions exceeding 5% CNT were not investigated in this work, as higher CNT loadings led to poor structural integrity of the electrode material in the dry state. The in the upper-right corner of Figure 1a shows a representative surface microstructure of the composite, acquired using a Zeiss Sigma300 (Zeiss, Jena, Germany) scanning electron microscope. Figure 1b–e present cross-sectional SEM images of the RGO-CNTs composites, revealing a relatively uniform porous architecture with well- interconnected networks formed between RGO sheets and CNTs. Figure 1f–i display cross-sectional views of pristine RGO, which also exhibits a porous morphology. However, at the same magnification, the RGO-CNTs composite shows a denser pore structure.

**Figure 2:**
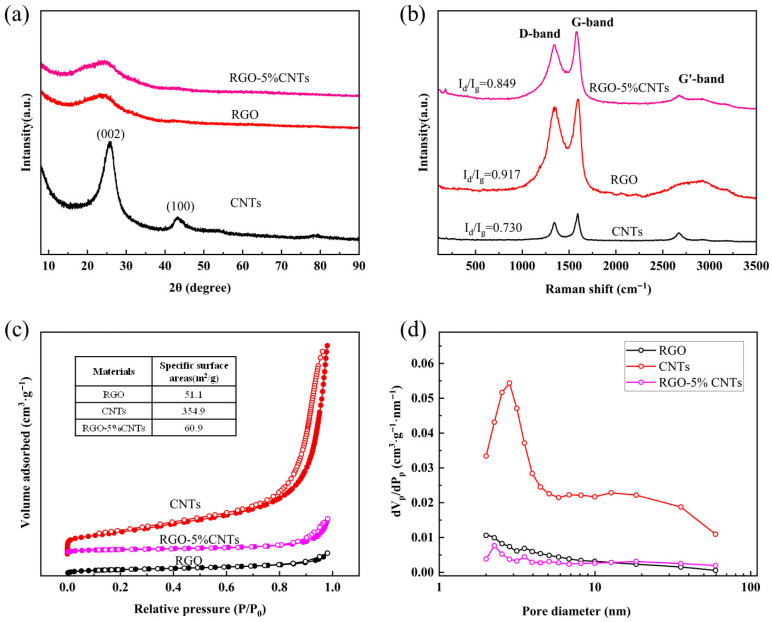
(**a**) XRD patterns, (**b**) Raman spectra, (**c**) nitrogen adsorption and desorption isotherm, and (**d**) BJH adsorption pore size distribution of the CNTs (black curve), RGO (red curve), and RGO-CNTs (purple curve), respectively. The full circles and the empty circles in (**c**) form isothermal adsorption curve and isothermal desorption curve, respectively.

The structure characteristics of RGO were investigated by X-ray diffraction (XRD) using a scanning step size of 0.05°/s over a 2*θ* range of 5° to 60°, with an operating voltage of 40 kV and a current of 30 mA. As shown by the black curve in Figure 2a, the XRD pattern of single-walled carbon nanotubes shows the highest diffraction intensity at 26°, corresponding to the (002) plane associated with interlayer stacking of conjugated aromatic structures. A weaker diffraction peak appears at 43°, assigned to the (100) plane of CNTs [41]. The XRD pattern of RGO exhibits a characteristic graphene peak at 2*θ* = 26°, indicating complete reduction in GO [42]. In the RGO-CNTs composite, the dominant peak at 26° reflects features common to both components, while a faint peak at 43° suggests the presence of a minor amount of CNTs. The XRD patterns of RGO and RGO-CNTs composites exhibit broadened diffraction peaks (002), primarily due to structural defects and interlayer disorder. The redox method for preparing RGO significantly disrupts the long-range ordered sp^2^ carbon lattice structure within the graphene sheets, resulting in a high degree of disorder. Residual lattice defects during the reduction process prevent the reduced RGO sheets from being perfect planes, leading to smaller crystalline domain sizes and peak broadening. In macroscopic powder samples, the random orientation of countless CNTs or CNT bundles and the disordered stacking of tubes directly contribute to the broadening of the (002) diffraction peak, corresponding to the inter-tube stacking plane, imparting a “semi-crystalline” character to the CNTs.

Figure 2b displays the Raman spectra of CNTs, RGO, and RGO-CNTs composite. The D and G bands, which are characteristic features in the Raman spectra of carbon nanostructures including graphene and carbon nanotubes, provide key insights into the structural and electronic properties of these materials. The G band corresponds to the E2g phonon mode, which is a fundamental in-plane vibration of the sp^2^-hybridized carbon atoms in the hexagonal lattice. The G band, appearing around 1580 cm^−1^ in the Raman spectra of carbon nanostructures [43], is commonly known as the ‘graphite’ band. It originates from the in-plane bond-stretching vibrations of C-C bonds in sp^2^-hybridized carbon networks. The intensity and sharpness of the G band reflect the graphitic ordering and crystallinity of the material, with higher and narrower peaks indicating a more ordered structure. In contrast, the D band—often termed the ‘disorder’ band—arises from breathing modes of k-point phonons associated with structural defects, edges, or lattice imperfections. Its intensity correlates directly with the density of defects in the carbon framework [44,45,46]. In this study, the ID/IG ratios for CNTs, RGO, and RGO-CNTs were measured as 0.730, 0.917, and 0.849, respectively. The higher ratio in RGO suggests a greater concentration of structural defects, while the intermediate value for the RGO-CNTs composite indicates that the incorporation of CNTs partially mitigates disorder. All three samples also exhibit the G’ (2D) band, which signifies the presence of defect-free sp^2^ carbon domains.

The specific surface area of the samples was evaluated by nitrogen adsorption measurements at 77 K using the BELSORP MaxII ASAP 2020 analyzer (MicrotracBEL, Osaka, Japan). Prior to analysis, each sample (approximately 200 mg) was degassed at 150 °C under secondary vacuum. Adsorption isotherms were collected over a relative pressure (*p*/*p*_0_) range of 0.001–0.99, followed by desorption from 0.99–0.1 *p*/*p*_0_. The Brunauer–Emmett–Teller (BET) method was applied to determine the specific surface area by linearizing the adsorption branch to obtain the monolayer nitrogen adsorption capacity. As shown in Figure 2c, the N_2_ adsorption–desorption isotherms for RGO, CNTs, and RGO-5%CNTs yield BET surface areas of 51.1 m^2^/g, 354.9 m^2^/g, and 60.9 m^2^/g, respectively. The corresponding pore size distribution curves (Figure 2d) reveal that all three materials are predominantly micropores. At first glance, the low SSA of the composite compared to the pristine CNTs appears counterintuitive. However, a critical analysis provides a plausible mechanism for this observation. The high SSA of the pristine CNTs (354.9 m^2^/g) primarily originates from their inner hollow channels and the inter-tubular spaces within the bundles. In contrast, the RGO sheets, despite their theoretical high surface area, often suffer from severe restacking during reduction, leading to the measured low SSA of 51.1 m^2^/g. We propose that during the composite formation process, the large, flexible RGO sheets intimately wrap around and encapsulate the CNT bundles. This wrapping phenomenon has two major consequences: (1) It effectively blocks the access of N_2_ molecules to the mesoporous network of the CNT bundles, which was the primary source of their high surface area. (2) The external surface of the composite becomes dominated by the relatively less porous RGO sheets. Consequently, the overall measured SSA of the composite is drastically reduced and approaches the value of the restacked RGO component. This structural model is supported by our TEM images (Figure 1a), which show a close association between the wrinkled RGO sheets and the CNTs. Despite the reduction in SSA, the composite exhibits superior thermoelectric performance (e.g., higher thermoelectric coefficient and low internal resistance as discussed in Section 3. This underscores that while SSA is an important factor, the synergistic effects such as enhanced electrical conductivity due to the CNT backbone and the prevention of RGO restacking are more critical for the final application performance. The composite architecture successfully balances these competing factors.

**Figure 3:**
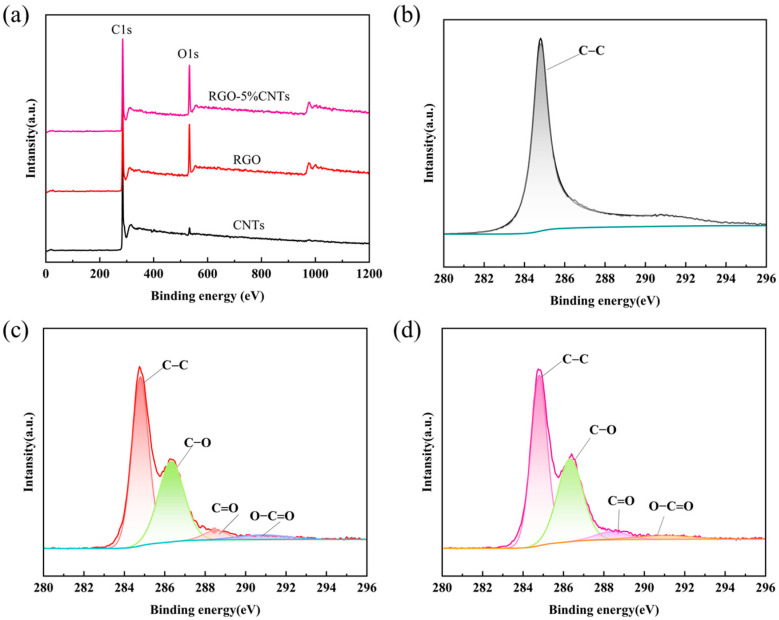
(**a**) XPS survey spectra of all elements (C 1s and O 1s) for CNTs (black curve), RGO (red curve), and RGO-CNTs (purple curve). XPS spectrum of C 1s for (**b**) CNTs, (**c**) RGO, and (**d**) RGO-CNTs. The green curve in (**b**), the blue curve in (**c**), and the orange curve in (**d**) are referred to as background curves, used for calibration and reference positioning.

To further elucidate the elemental composition and chemical bonding states of the composites, X-ray Photoelectron Spectroscopy (XPS) was performed, as summarized in Figure 3. The survey scans of RGO and RGO–5%CNT over a binding energy range of 0–1200 eV display two dominant peaks corresponding to C1s and O1s (Figure 3a). As shown in Figure 3b, the C1s spectrum of CNTs is dominated by a single component at 284.80 eV, attributed to aromatic or conjugated carbon. In contrast, the high-resolution C1s spectra for both RGO and RGO-5%CNT (Figure 3c,d) can be deconvoluted into five distinct contributions, representing carbon in different chemical environments: aromatic/conjugated C (284.80 eV), C–O–C (286.40 eV), O=C–C (~288.6 eV), and O–C=O, 289.00 eV [42,47].

**Figure 4:**
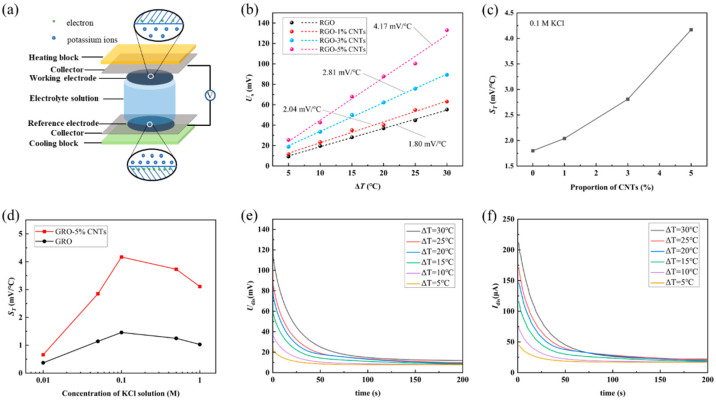
(**a**) Schematic diagram of the thermoelectric conversion cell. (**b**) Relation of equilibrium voltage *U*_s_ and the temperature difference Δ*T* of RGO, RGO-1%CNTs, RGO-3%CNTs, and RGO-5%CNTs. The dashes in the figure are linear fitting curves. (**c**) Relationship between thermoelectric coefficient and change in CNT content of electrode material in the thermoelectric conversion cell with 0.1 M KCl. (**d**) Relationship between thermoelectric coefficient and concentration of KCl solution of thermoelectric conversion cell with RGO and RGO-5%CNTs electrodes. (**e**) Discharging voltage over the loading resistance in different temperature difference. (**f**) Discharging current among the loading resistance in different temperature difference.

**Thermoelectric Cell Assembly.** The schematic diagram of the thermoelectric conversion cell is shown in Figure 4a. Ions electrostatically adsorb on the surface of the electrode, forming a double-layer structure, which results in the generation of a potential on the graphene surface. Previous molecular dynamics simulations have indicated that the ion distribution within the electrical double layer between potassium chloride solution and graphene varies with temperature [48]. Differences in the double-layer distribution induce a potential difference between the surfaces of graphene electrodes at different temperatures. This potential difference, as the open-circuit voltage of a thermoelectric conversion cell, can be measured using a digital multimeter (Fluke 8808A, Everett, WA, USA). When a load resistor is connected between the graphene electrodes, electrons will move from the lower potential side to the higher potential side under the influence of the electric field. This process represents the conversion of thermal energy into electrical energy by the thermoelectric conversion cell. During cell assembly, the RGO-CNTs was first bonded to a stainless-steel current collector using conductive silver paste to form the working/reference electrode. The electrode was then mounted onto a heating/cooling plate and embedded at both ends of a polytetrafluoroethylene (PTFE) tube. The entire assembly was mechanically reinforced with steel plates and bolts before the electrolyte was introduced via syringe until the working and reference electrodes were fully immersed. After a certain period, the ionic distribution near the electrode surface progressively approached equilibrium, as indicated by the gradual stabilization of the open-circuit voltage between the two current collectors close 0 mV. A temperature sensor was attached to the current collector to monitor the approximate temperature of the electrode during operation. The fully assembled thermoelectric cell was placed on an electrically insulated platform to minimize external interference.

## 3. Results & Discussion

**The output voltage and thermoelectric coefficient of the thermoelectric conversion cell.** Upon applying a temperature gradient across the thermoelectric conversion cell, variations in the EDL structure-induced by thermal effects in the electrode materials and potassium chloride (KCl) electrolyte lead to measurable changes in the cell’s output voltage. The study employed four types of electrode materials: RGO, RGO-1%CNTs, RGO-3%CNTs, and RGO-5%CNTs. A series of KCl solutions with concentrations of 0.01 M, 0.05 M, 0.1 M, 0.5 M, and 1 M were injected into the cell. The cold side was maintained at 15 °C via a cooling water circulation system, while the hot side was set to temperatures of 20 °C, 25 °C, 30 °C, 35 °C, 40 °C, and 45 °C, corresponding to temperature differences Δ*T* of 5 °C, 10 °C, 15 °C, 20 °C, 25 °C, and 30 °C, respectively. The output voltage *U*_out_ between the working electrode and reference electrodes, together with the electrode temperature, was continuously monitored. It was observed that *U*_out_ began to rise from 0 mV as soon as Δ*T* was imposed. After Δ*T* reached the target value and stabilized, *U*_out_ continued to increase for a period before attaining a steady-state voltage *U*_s_, indicating that the ionic distribution at the electrode–electrolyte interface requires finite time to equilibrate under the new thermal condition. Conversely, as Δ*T* decreased, *U*_out_ dropped almost synchronously and eventually returned to near 0 mV after Δ*T* was maintained at 0 °C for a certain duration (for further details, see our previous report [49]).

As shown in Figure 4b, a nearly linear relationship is observed between the equilibrium voltage (*U*_s_) and the temperature difference (Δ*T*) for different electrode materials incorporated in the thermoelectric conversion cell. There is an approximately linear relationship between the equilibrium voltage. Linear fitting of the experimental *U*_s_ versus Δ*T* data yields slopes of 1.80 mV/°C for RGO, 2.04 mV/°C for RGO-1%CNTs, 2.81 mV/°C for RGO-3%CNTs, and 4.17 mV/°C for RGO-5%CNTs. These slopes share the same unit as the Seebeck coefficient and reflect the electric potential response of the cell to thermal energy; they are therefore defined as the thermoelectric coefficient *S_T_* of the cell. The value of *S_T_* is influenced by multiple factors, such as the type and concentration of electrode materials, electrolyte composition, solvents, and the cell configuration. This study focuses specifically on the effects of electrode materials and KCl concentration on *S_T_*. Figure 4c illustrates the relationship between *S_T_* with higher CNT content. Owing to limitations in the current electrode fabrication process, electrodes with CNT contents beyond 5% were not experimentally accessible. Given the high specific surface area and developed microporous structure of CNT-based materials, the thermoelectric coefficient of pure CNTs films may exhibit potential-dependent behavior. Thus, future work will focus on developing fabrication methods for freestanding CNT films and systematically characterizing their thermoelectric properties.

An analysis of the experimental results reveals the relationship between the thermoelectric coefficient *S_T_* and the concentration of KCl solution, as shown in Figure 4d. It is found that as the concentration of KCl solution increases, the thermoelectric coefficient *S_T_* first increases and then decreases. After the KCl solution concentration exceeded 0.1 M, a significant decrease in the ST value was observed. This phenomenon can be rationalized by considering the electrostatic shielding effect induced by the high ionic concentration of the KCl solution [50]. When the KCl concentration is below 0.1 M, the electrode material surface possesses a certain surface charge (for example, RGO carries a negative charge), which allows the formation of a well-developed electric double layer (EDL). However, when high concentrations of KCl (greater than 0.1 M) are introduced, the abundant K^+^ and Cl^−^ ions effectively screen the surface charge of the material. This compresses the electric double layer and significantly weakens the long-range electrostatic forces. Consequently, the nature of interactions at the interface changes. The weakening of electrostatic repulsion may allow [K^+^ and Cl^−^] ions to approach the surface more closely or exist in different orientations, which alters the surface potential of the electrode material and ultimately leads to a decrease in the observed ST values. Similar ion-concentration-dependent effects on surface charge have also been reported in the literature [51,52]. To enhance the reproducibility of the experiments, all the above results are averages obtained from three measurements.

**Discharge characteristics of thermoelectric conversion cells.** When the working electrode is connected to the reference electrode via an external load resistance *R*_0_, a closed circuit is established. Electrons accumulated at the electrode–electrolyte interface then flow through the external path, resulting in the discharge of the EDL and generating a measurable current. This process represents the discharge behavior of a thermoelectric cell. As the system approaches a new steady state, both the discharge voltage *U*_dis_ across *R*_0_ and the discharge current *I*_dis_ through it stabilize. As shown in Figure 4e,f, for a cell using 0.1 M KCl and an RGO–5%CNTs electrode, *U*_dis_ and *I*_dis_ exhibit rapid decay followed by stabilization over time. This behavior suggests that as the EDL capacitance discharges, continuous ion migration and electron replenishment at the electrode–electrolyte interface sustain a dynamic equilibrium. Such a mechanism implies that under a maintained temperature difference, the EDL can provide sustained power output to the external circuit over extended periods. The detailed dynamics of EDL evolution under thermal gradients are complex and will be the focus of our subsequent investigations.

**Figure 5:**
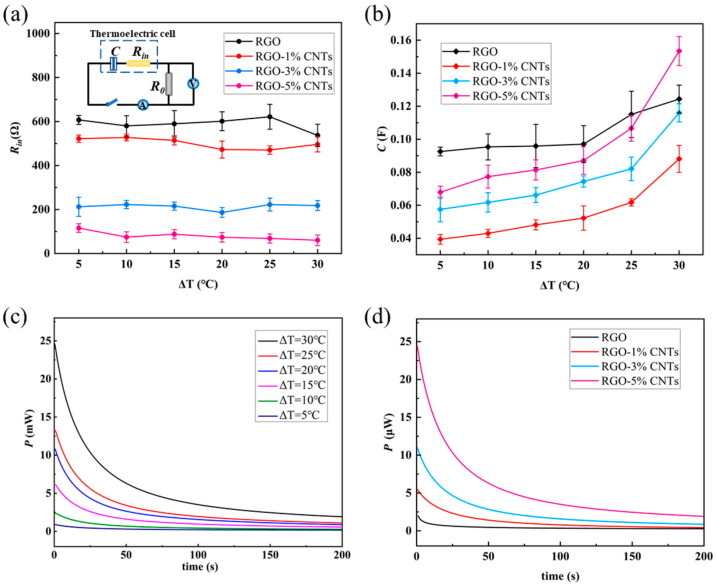
(**a**) The internal resistance *R*_in_ and (**b**) capacitance *C* of thermoelectric conversion cells with 0.1 M KCl solution of RGO, RGO-1%CNTs, RGO-3%CNTs, RGO-5%CNTs electrodes. The inset in (**a**) is the equivalent circuit. The error bars represent the standard deviation. (**c**) The instantaneous output power *P* of thermoelectric conversion cells with 0.1 M KCl solution and RGO-5%CNTs electrodes of different Δ*T*. (**d**) The instantaneous output power *P* of thermoelectric conversion cells with 0.1 M KCl solution of RGO, RGO-1%CNTs, RGO-3%CNTs, RGO-5%CNTs electrodes.

If the thermoelectric conversion cell is modeled as a series circuit consisting of a capacitor (capacitor *C*) and a resistor (internal resistance *R*_in_), as illustrated in Figure 5a, the values of *R*_in_ and *C* can be determined using the following expression

(1) $$R_{\mathrm{in}}=U_{s}/I_{\max}-R_{0}$$

(2) $$C=Q/\Delta U_{\mathrm{dis}}=\int Idt/(U_{s}-0)=\int Idt/U_{s}$$

where *I*_max_ is the maximum of *I*_dis_, *Q* refers to the charge transferred during the discharge process, and Δ*U*_dis_ is the total change in discharge voltage.

As shown in Figure 5a, the internal resistance *R*_in_ is only weakly influenced by the temperature difference but decreases noticeably with increasing CNT content in the electrode material. This behavior can be attributed to two contributing factors: first, the incorporation of CNTs improves electrical connectivity between RGO sheets, enhancing the overall electronic conductivity of the electrode; second, CNTs facilitate ion transport within the electrolyte, thereby reducing charge transfer resistance. Meanwhile, the equivalent capacitance *C*, presented in Figure 5b, increases consistently with the applied temperature difference Δ*T*. The black data points in Figure 5b represent the capacitance values corresponding to the RGO electrodes. With the addition of CNT material, the capacitance of the cell with the RGO-1%CNTs electrode significantly decreases. When the CNT content is further increased (RGO-3%CNTs and RGO-5%CNTs), the capacitance of the cell increases accordingly. This non-monotonic behavior can be explained by the continuously changing interaction between two key factors: conductivity and effective ionic contact area. In the region of low CNT content (for example, from 0% to 1%), incorporating a small amount of CNT is insufficient to form a percolation network for efficient electron transport. At this stage, the highly overlapping RGO dense layered structure severely hinders the penetration and diffusion of electrolyte ions, resulting in a large surface area being electrochemically inactive. Therefore, the overall capacitance is low. However, when the CNT content exceeds a critical threshold (>1%), a synergistic effect occurs. The CNTs begin to intertwine with RGO sheets, forming a three-dimensional (3D) hierarchical conductive structure. This structure plays a dual role: first, the rigid CNTs act as nanoscale spacers, effectively slowing down the restacking of RGO sheets, creating more nanopores and ion transport channels, thereby increasing the EDL region. Second, the continuous CNT network provides pathways for rapid electron collection and transport, significantly reducing the internal resistance of the electrode. The enhanced ion and electron transport capabilities together lead to a significant recovery and eventual increase in capacitance.

Further analysis of the discharge voltage and discharge current curves allows for the calculation of the instantaneous output power *P* of the circuit using the formula below

(3) $$P=\int \Delta U_{\mathrm{dis}}^{2}/R_{0}dt/\Delta t$$

where Δ*t* is the discharging time. Figure 5c,d present the instantaneous output power as a function of time for electrodes with different CNT contents under varying temperature gradients. The output power decays gradually over time before eventually stabilizing. Both higher temperature differences and increased CNT content lead to greater initial output and a more rapid decay rate.

**Figure 6:**
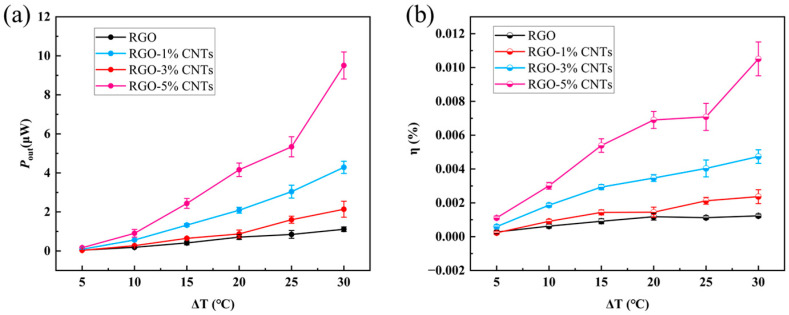
(**a**) The average output power *P* and (**b**) energy conversion efficiency *η* of the thermoelectric conversion cells with 0.1 M KCl solution of RGO, RGO-1%CNTs, RGO-3%CNTs, RGO-5%CNTs electrodes. The error bars represent the standard deviation.

**The efficiency of thermoelectric conversion cells.** To further evaluate the energy conversion performance of thermoelectric conversion cells, the average output power *P*_out_ was analyzed. Taking into account the temporal decay of the discharge voltage, the time interval during which the voltage decays to 30% of the equilibrium voltage *U*_s_ was used to calculate *P*_out_ according to Equation (3). As shown in Figure 6a, the average output power increases with both the temperature difference and the CNT content in the electrode. The input power and energy conversion efficiency were evaluated following the method reported in our previous work [49]. Figure 6b presents the energy conversion efficiency *η* of the thermoelectric cell with 0.1 M KCl solution. The value of *η* rises with increasing temperature difference and CNT content. At a temperature difference of 30 °C, the calculated efficiencies for RGO, RGO-1%CNTs, RGO-3%CNTs, and RGO-5%CNTs electrodes are 0.0012%, 0.0024%, 0.0047%, and 0.011%, respectively. Increasing the CNT content from 0% to 1% doubles the efficiency, while a CNT content of 5% leads to an order-of-magnitude enhancement compared to pure RGO. These results clearly demonstrate that the incorporation of CNTs significantly improves the thermoelectric conversion performance of RGO-based cells, underscoring the potential of carbon nanotubes in low-grade thermal energy harvesting.

**Figure 7:**
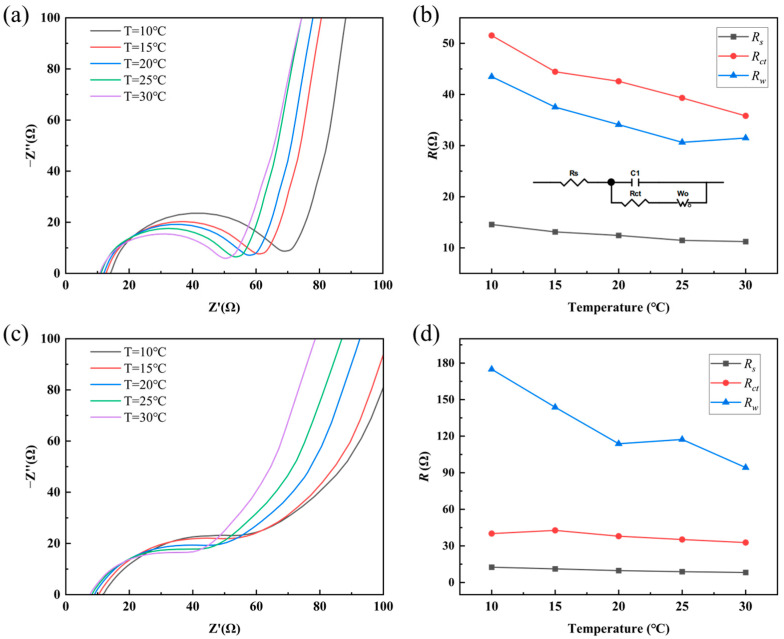
EIS plots of (**a**) RGO-5%CNTs and (**c**) RGO immersed in 0.1 M KCl solution under temperature of 10 °C, 15 °C, 20 °C, 25 °C, 30 °C. *R*_s_, *R*_ct_, and *R_w_* of (**b**) RGO-5%CNTs and (**d**) RGO immersed in 0.1 M KCl solution.

In the preceding analysis, the thermoelectric cell was modeled as a series circuit consisting a capacitor (capacitance *C*) and a resistor (internal resistance *R*_in_) for the qualitative investigation of the effects of solution concentration and temperature. To better understand the energy loss mechanisms in thermoelectric cells during discharge—which is essential for subsequent efficiency analysis—a more refined equivalent circuit model is required. For comparing the charge transfer behavior of electrode materials in 0.1 M KCl solution at different temperatures, Electrochemical Impedance Spectroscopy (EIS) was performed on RGO films and RGO-5%CNT composites, as shown in Figure 7a,c. In all EIS tests conducted in this study, the initial voltage was 0 V, the high frequency was 1 × 10^5^ Hz, the low frequency was 0.01 Hz, and the voltage amplitude was 0.005 V. Based on the test results of EIS, the solid–liquid system consisting of electrode material and KCl solution can be represented by the equivalent circuit shown in Figure 7b. Here, *R*_s_ represents the solution resistance, *R*_ct_ the charge transfer resistance, *C* the double-layer capacitance, and *R_w_* the Warburg diffusion impedance. Fitting of the EIS data yielded the values of *R*_s_, *R*_ct_, and *R_w_* for RGO and RGO-5%CNTs, as summarized in Figure 7b,d. With increasing temperature, *R*_s_, *R*_ct_, and *R_w_* all decrease gradually, indicating that elevating the temperature effectively reduces the internal impedance of the thermoelectric cell. It is worth noting that the values of *R*_s_ and *R*_ct_ are similar for both electrode materials. However, the Warburg diffusion impedance *R_w_* of the RGO-5%CNTs composite is approximately one-third of that for the pristine RGO, suggesting that the incorporation of CNTs facilitates ion diffusion within the nanoporous structure of the electrode. This enhanced ionic transport contributes to lower energy losses and improved energy conversion efficiency of the cell. In addition, Table 1 presents the capacitance values of capacitor C1 in the equivalent circuit at different temperatures. Overall, the capacitance values of the RGO-5%CNTs electrode and the RGO electrode are quite similar, with the RGO-5%CNTs electrode exhibiting a slightly higher average capacitance across different temperatures.

**Discussion on the Enhanced Thermoelectric Mechanism in RGO-5%CNTs Composite.** The remarkable thermoelectric performance of the RGO-5%CNTs composite, as evidenced by its higher output voltage and power (Figure 4b and Figure 6a), can be understood through its hierarchical structure and the underlying mechanism. As described in the Methods, the fundamental principle involves a temperature-gradient-induced potential difference across the EDL at the electrode-electrolyte interface [48]. In the RGO-5%CNTs composite, this process is significantly enhanced by two key synergistic effects. First, while the pristine RGO sheets provide a high-surface-area substrate for ion adsorption and EDL formation, their semiconducting nature can limit electron transport. The incorporation of 5% CNTs creates a highly conductive percolating network that acts as an “electron highway”, enabling efficient collection and transport of compensating charges from the graphene surface to the external circuit with minimal resistance. This accounts for the observed reduction in the cell’s internal resistance (Figure 5), leading to higher output current and power. Second, the CNTs interspersed between the RGO sheets function as nano-spacers, mitigating RGO restacking and preserving an open porous structure. This facilitates electrolyte ion infiltration and efficient diffusion, which is essential for rapid EDL formation and modulation under a thermal gradient. A more robust and accessible EDL enables more effective and stable thermovoltage generation. In summary, the RGO-5%CNTs composite retains the fundamental EDL-based mechanism but optimizes its efficacy. The CNT network enhances electronic conduction for efficient charge delivery while improving ionic accessibility for effective EDL formation, collectively leading to the superior thermoelectric conversion performance reported.

**Discussion on the Electrode Processes.** Inspired by the work of [53] on similar carbon-based systems, we propose a plausible description of the electrode processes governing the thermoelectric effect in our RGO-CNTs-based cell. The core mechanism likely involves the entropy-driven thermodiffusion (Soret effect) of ions in the temperature gradient and their subsequent interaction with the electrode-electrolyte interface. When a temperature difference (Δ*T*) is established between the two electrodes, a thermal field is superimposed on the thermoelectric cell. This field drives the differential migration of K^+^ and Cl^−^ ions due to their distinct ionic Seebeck coefficients. Typically, cations and anions exhibit different mobilities and solvation entropies in a thermal gradient, leading to their spatial separation. The selective accumulation of ionic species (e.g., an excess of one ion type at the hot electrode) disrupts the charge neutrality at the electrode-electrolyte interface. This induces an equal and opposite electronic charge on the electrode surface to maintain electrostatic equilibrium, thereby modulating the structure and potential of the EDL. The high surface area and rich oxygen-containing functional groups of the RGO-CNTs composite are critical here, as they provide abundant sites for ion adsorption and efficient EDL formation. The differential charging of the hot and cold electrodes establishes a steady-state thermovoltage (*U*_s_). This potential counteracts the continued ion flux until a dynamic equilibrium is reached. The process can be viewed as a thermally charged supercapacitor, where the temperature gradient performs the work of ‘charging’ the EDL. Upon connecting an external load, the stored capacitive energy is released. The discharge profile (rapid decay followed by stabilization) reflects the complex interplay between the internal resistance (*R*_in_), dominated by the electrode’s electronic conductivity and the electrolyte’s ionic resistance, and the equivalent capacitance (*C*), which represents the total charge stored in the EDL. The enhancement of this process by CNTs can be attributed to their role in facilitating faster electron transport across the electrode, thereby improving charge collection and delivery to the external circuit.

**Figure 8:**
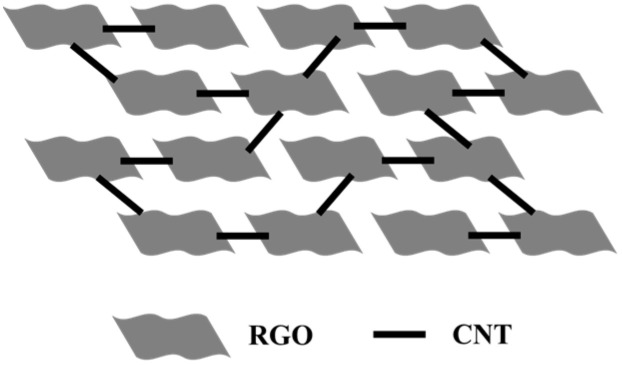
Hierarchical structural model of RGO-CNTs composites.

**Mechanistic Discussion on the Synergistic Network.** The superior performance of the RGO-CNTs composites can be understood through a qualitative model of their hierarchical structure, as illustrated in Figure 8. In this framework, the two-dimensional RGO sheets provide a continuous, high-surface-area scaffold for ion adsorption and thermal charging, while the one-dimensional CNTs are intricately woven into this network, creating a synergistic effect. Firstly, regarding charge transport, the CNTs act as “electronic highways” that penetrate the RGO sheets. The semi-conducting nature of RGO often leads to high intrinsic resistance. The incorporation of highly conductive CNTs forms a percolating network that drastically reduces the tortuosity for electron flow. This direct, low-resistance pathway is crucial for efficiently collecting the thermally generated charges and delivering them to the external circuit, thereby minimizing parasitic losses and enhancing the output power. Secondly, concerning heat diffusion, the same CNT network also serves as a “thermal conduit”. Although not directly measured here, it is well-established that CNTs possess exceptionally high axial thermal conductivity [54]. These CNT bridges facilitate rapid heat distribution throughout the composite, preventing the formation of localized hot spots and ensuring a more uniform temperature gradient across the electrode. This efficient heat management is essential for maintaining a stable thermovoltage and improving the device’s robustness. The synergy arises from the fact that a single structural component (the CNT network) simultaneously optimizes both electrical and thermal transport. The composition and microstructure are therefore critical: at low CNT content, the network is incomplete, and the benefits are marginal. At an optimal loading (e.g., 5%), a continuous, interpenetrating network is formed, maximizing the synergistic effect without causing excessive agglomeration that could compromise the accessible surface area of the RGO. This structural optimization directly translates to the peak device efficiency observed in our experiments”.

## 4. Conclusions

In this work, we successfully fabricated a self-assembled RGO-CNTs composite via a redox and freeze-drying approach. The composite structure synergistically enhanced the material’s thermoelectric conversion capabilities: the RGO framework provided a microporous network for ion transport and active sites for thermal charging, while the incorporated CNTs drastically improved electrical conductivity and served as critical structural spacers. The key finding is that the CNT content is a pivotal factor in optimizing performance. An increase in CNTs led to a consistent enhancement of the thermoelectric coefficient and a significant reduction in the cell’s internal resistance. Notably, a non-monotonic relationship was observed for the equivalent capacitance, suggesting an optimal balance between improved conductivity and accessible surface area. Consequently, the RGO-5%CNTs composite delivered a remarkable, order-of-magnitude improvement in energy conversion efficiency compared to pure RGO. This study demonstrates that rational structural engineering of carbon-based composites is a promising strategy for developing high-performance thermoelectric conversion cells, providing valuable insights for future design of thermal energy harvesting devices.

Finally, while the RGO-CNTs composites demonstrate promising initial performance, future work should include a comprehensive investigation into their long-term operational stability under continuous thermal cycling and the associated aging mechanisms. A detailed investigation of the thermo-mechanical properties of these composites also represents an important direction for future research, particularly for applications requiring mechanical flexibility or exposure to extreme temperatures.

## Figures and Tables

**Table 1:** Capacitance of C1 variation with temperature.

Temperature (°C)	C1 (F)	
	RGO-5%CNTs	RGO
10	1.1546 × 10^−4^	9.9655 × 10^−5^
15	1.1786 × 10^−4^	1.0230 × 10^−4^
20	1.1432 × 10^−4^	1.0758 × 10^−4^
25	1.1083 × 10^−4^	1.1217 × 10^−4^
30	1.0869 × 10^−4^	1.1452 × 10^−4^

## Data Availability

The original contributions presented in this study are included in the article. Further inquiries can be directed to the corresponding authors.
